# Ambient nitrogen dioxide is associated with emergency hospital visits for atrial fibrillation: a population-based case-crossover study in Reykjavik, Iceland

**DOI:** 10.1186/s12940-021-00817-9

**Published:** 2022-01-03

**Authors:** Solveig Halldorsdottir, Ragnhildur Gudrun Finnbjornsdottir, Bjarki Thor Elvarsson, Gunnar Gudmundsson, Vilhjalmur Rafnsson

**Affiliations:** 1 University of Iceland, Centre of Public Health Science, Reykjavik, Iceland; 2 Environment Agency of Iceland, Reykjavik, Iceland; 3 Marine and Freshwater Research Institute, Reykjavik, Iceland; 4 Faculty of Medicine, University of Iceland, Reykjavik, Iceland; 5 Department of Respiratory Medicine & Sleep, Landspitali University Hospital, Reykjavik, Iceland; 6 University of Iceland, Department of Preventive Medicine, Reykjavik, Iceland

**Keywords:** Nitrogen dioxide, Atrial fibrillation, Cardiac arrhythmia, Ischemic heart diseases, Population-based, Hospital registry, Case-crossover

## Abstract

**Background:**

In Iceland air quality is generally good; however, previous studies indicate that there is an association between air pollution in Reykjavik and adverse health effects as measured by dispensing of medications, mortality, and increase in health care utilisation.

The aim was to study the association between traffic-related ambient air pollution in the Reykjavik capital area and emergency hospital visits for heart diseases and particularly atrial fibrillation and flutter (AF).

**Methods:**

A multivariate time-stratified case-crossover design was used to study the association. Cases were those patients aged 18 years or older living in the Reykjavik capital area during the study period, 2006–2017, who made emergency visits to Landspitali University Hospital for heart diseases. In this population-based study, the primary discharge diagnoses were registered according to International Classification of Diseases, 10th edition (ICD-10). The pollutants studied were NO_2_, PM_10_, PM_2.5_, and SO_2,_ with adjustment for H_2_S, temperature, and relative humidity. The 24-h mean of pollutants was used with lag 0 to lag 4.

**Results:**

During the study period 9536 cases of AF were identified. The 24-h mean NO_2_ was 20.7 μg/m^3^. Each 10 μg/m^3^ increase in NO_2_ was associated with increased risk of heart diseases (ICD-10: I20-I25, I44-I50), odds ratio (OR) 1.023 (95% CI 1.012–1.034) at lag 0. Each 10 μg/m^3^ increase in NO_2_ was associated with an increased risk of AF (ICD-10: I48) on the same day, OR 1.030 (95% CI: 1.011–1.049). Females were at higher risk for AF, OR 1.051 (95% CI 1.019–1.083) at lag 0, and OR 1.050 (95% CI 1.019–1.083) at lag 1. Females aged younger than 71 years had even higher risk for AF, OR 1.077 (95% CI: 1.025–1.131) at lag 0. Significant associations were found for other pollutants and emergency hospital visits, but they were weaker and did not show a discernable pattern.

**Conclusions:**

Short-term increase in NO_2_ concentrations was associated with heart diseases, more precisely with AF. The associations were stronger among females, and among females at younger age. This is the first study in Iceland that finds an association between air pollution and cardiac arrhythmias, so the results should be interpreted with caution.

**Supplementary Information:**

The online version contains supplementary material available at 10.1186/s12940-021-00817-9.

## Introduction

In a review of epidemiological studies on air pollution and hospital admissions, exposure to several air pollutants was found to be associated with cardiovascular diseases (CVD) [1]. Since the publication of this review, CVD have been associated with air pollution in recent studies from developing countries [2, 3] as well as in developed countries [4, 5]. In these studies, the outcome of CVD has been broadly defined [2] or attributed to acute myocardial infarction [3] and a range of cardiovascular events [5]. Cardiac arrhythmias are among the cardiovascular events found to be in association with air pollution [6], and atrial fibrillation (AF) has been associated with ambient NO_2_ and particulate air pollution [5, 7–9]. However, in some studies no association between exposure to air pollution and AF onset was found [10] or a weak association between NO_2_ exposure and AF was observed [11], and in this context, the type and magnitude of the involved pollutants and the statistical power of the studies have been discussed [9].

A recent multilocation analysis of associations between a short-term increase in NO_2_ and daily total, cardiovascular, and respiratory mortality did not lack statistical power [12]. This study found that an increase in NO_2_ on the previous day was significantly associated with increased risk of cardiovascular mortality [12], which immediately raises the question of which condition of this broad disease category is of practical importance. Currently, indications of an increase of AF have been found in studies from the US [13] as well as Iceland [14] where the prevalence was 2% in the year 2008.

The setting of Reykjavik, Iceland, offers an opportunity to study the association between air pollution and adverse health effects in a temperate/cold climate zone with relatively low daily mean pollution due primarily to particulate matter and NO_2_ originating from traffic [15, 16]. Previous studies in the Reykjavik capital area indicate an association between air pollution and adverse health effects as measured in the form of increased mortality, medication dispensing for asthma and angina pectoris, and emergency hospital admissions [17–21]. In Reykjavik, daily exposure to PM_10_, PM_2.5,_ and NO_2_ is substantially lower than in many of the above-mentioned studies [3, 5, 6, 9], but on par with the lowest reported exposures [7, 11].

The aim of this study was to evaluate the association between traffic-related pollution (NO_2_, PM_10_, PM_2.5,_ and SO_2_) in the Reykjavik capital area and emergency hospital visits for heart diseases and in particular AF as the primary discharge diagnosis. Results were adjusted for H_2_S emissions from geothermal/industrial sources and meteorological variables.

## Methods

### Site description and study base

The Reykjavik capital area is in the southwestern part of Iceland and is the northernmost capital in the world. Traffic is the main source of air pollution in the city, and other sources include two geothermal power plants, Hellisheidi which opened in September 2006 (located 26 km east-southeast of the city), and the smaller Nesjavellir which opened in 1990 (located 33 km east of the city). Reykjavik’s capital area spreads over 247.5 km^2^ and in 2017 the inhabitants numbered 217,000, equivalent to two-thirds of the total Icelandic population [22]. The study base included the residents of the greater capital area which includes seven municipalities (Gardabaer, Hafnarfjordur, Kjosarhreppur, Kopavogur, Mosfellsbaer, Reykjavik, and Seltjarnarnes) identified by 24 postal codes. The study period was January 1st, 2006 to December 31st, 2017. The annual mean population of the Reykjavik capital area during the study period was 203,500 [22].

### Study population

Hospital data were obtained from SAGA (Register of hospital-treated patients in Iceland) for all emergency department (ED) visits and acute admissions to Landspitali University Hospital (LUH) in the study period. LUH is operated by the Icelandic government and is the only acute care hospital in the Reykjavik capital area, making this study population-based. In Iceland, the national health insurance scheme is covered by taxes and available to all residents. Patients pay certain fees for ambulatory visits while admissions to the hospital are free of charge. The study population included adult inhabitants (≥ 18 years) of the Reykjavik capital area. At LUH diseases are classified and registered according to the International Classification of Diseases 10th edition (ICD-10). The cases had made an ED visit or were admitted to an inpatient ward of LUH during the study period and the primary discharge diagnoses were registered as certain heart diseases according to the ICD-10 codes: I20-I25, I44-I50. The outcomes analysed were heart diseases (ICD-10 codes: I20-I25, I44-I50), ischemic heart diseases (IHD) (I20-I25), cardiac arrhythmias and heart failure (I44-I50), and AF (I48). Readmissions within 10 days with the same ICD-10 primary discharge diagnosis were excluded. ED visits and acute hospital admissions were combined and will from now on be called emergency hospital visits.

### Air pollution data

Pollution data was obtained from Grensas measurement station (GRE), operated by the Environment Agency of Iceland. GRE is located in the centre of the Reykjavik capital area near one of the busiest road intersections in the city. Other measurement stations in the city did not have continuous measurements or permanent locations throughout the study period and were therefore not used in the study. However, to test if GRE was representative of the total capital area, Pearson’s correlation was calculated for GRE measurements and measurements from another station located in Dalsmari, Kopavogur municipality, for the period 2014–2017. Results of Pearson’s correlation coefficients between these two measurement stations were r = 0.44 to 0.98, depending on pollutants.

Pollutants measured at GRE were nitrogen dioxide (NO_2_), particulate matter less than 10 μm in diametre (PM_10_), particulate matter less than 2.5 μm in diametre (PM_2.5_), sulphur dioxide (SO_2_), and hydrogen sulphide (H_2_S) all measured in μg/m^3^. The meteorological data was obtained from the Icelandic Meteorological Office and included temperature (°C) and relative humidity (RH). PM_10_ was measured with an Andersen EMS IR Thermo (model FH62 I-R), NO_2_ with Horiba device (model APNA 360E), and SO_2_ and H_2_S with the Horiba model APOA 360E. Every 6–12 months the devices are calibrated. Exposure data included 12 years or 4383 days. Daily averages (midnight to midnight the following day) were calculated from hourly concentrations if at least 75% of one-hour data existed. Missing daily averages for NO_2_, PM_10_, PM_2.5_, SO_2_, and H_2_S were 383 days (8.7%), 165 days (3.8%), 923 days (21.1%), 200 days (4.6%), and 284 days (6.5%), respectively. Data gaps were seen, attributed to unknown reasons of inactive measurement devices, except for 52 days missing of H_2_S measurements at the beginning of the study period due to the fact that H_2_S measurements at GRE started at the end of February 2006. For temperature and RH, 6 days (0.1%) and 6 days (0.1%) were missing, respectively. Minor gaps in the curves were fitted by linear interpolation.

Descriptive statistics were calculated and showed as daily concentration levels in μg/m^3^ of the pollutants, and Spearman’s correlation test was used to analyse the trend in daily levels of pollutants through the study period.

### Design and data analysis

A time-stratified case-crossover design was used to estimate the association between daily exposure to air pollution and emergency hospital visits for heart disease. The study period was divided into monthly strata. Exposure during case periods (24 h) was compared to exposure during control periods, which were matched as the same weekdays within the same month (3–4 control periods per case period) [23, 24]. Several calculations were done: single pollutant models were calculated as well as multivariate models, containing all the traffic-related pollutants, H_2_S, temperature, and RH. Separate analyses were conducted for subgroups according to gender and age (≥ 71 and < 71 years). Furthermore, as sensitivity analysis, the data was restricted to ED visits only. Conditional logistic regression was used with adjusted odds ratios (OR), and 95% confidence intervals (CI) were calculated for every 10 μg/m^3^ increase of pollutants (24-h concentrations).

Five lag days (24 h) were analysed separately. The definitions of lags are as follows: lag 0: air pollution exposure on the same day as an emergency hospital visit, lag 1 to 4: air pollution exposure 1 day before (lag 1), and up to 4 days before (lag 4) the emergency hospital visit. As single-day lag models may underestimate these associations, we performed calculations of associations with 2-day (lag 0–1), and 3-day (lag 0–2) moving average of pollutants concentrations. The results of the multivariate models are presented in this article, and other results are shown in Tables C, and D in the Additional file 1.

Statistical analysis was done with R version 4.0.3 (https://www.r-project.org/). Statistical tests used in this study were all two-tailed and we considered results statistically significant for *p* < 0.05. The study was approved by the National Bioethics Committee (ref. no. VSNb2018120011/03.01), the Data Protection Authority (ref. no. 10–050), and the Scientific Committee of LUH.

## Results

Over the study period, there were 29,169 emergency hospital visits for the heart disease diagnoses included in the study (6.7 visits per day on average), a total of 13,664 individuals (40.1% females and 59.9% males) (Table 1). The median age was 71 years and the visits were divided into older (≥ 71 years) and younger (< 71 years) according to the median age (Table 1). The mean age for all heart disease visits was 68.7 years. On average, female patients were 4.5 years older than males during hospital visits. Of the total visits, 20,690 were ED visits while 8479 were acute admissions to inpatient wards.

**Table 1:** Descriptive statistics of emergency hospital visits for heart disease to Landspitali University Hospital, according to primary discharge diagnosis, January 1st, 2006 to December 31st, 2017

Discharge diagnosis	No. of visits	Visits per day					No. of patients
(ICD-10)	(%)	Mean (SD)	Range	P (25)	Median	P (75)	
Heart diseases (I20-I25, I44-I50)	29,169 (100)	6.69 (3.12)	0–22	4	6	9	13,664
Females	11,694 (40.1)	2.93 (1.68)	0–11	2	3	4	5718
Males	17,475 (59.9)	4.13 (2.23)	0–16	2	4	5	7946
Older (≥71 yr)	14,656 (50.2)	3.55 (1.99)	0–17	2	3	5	6556
Younger (< 71 yr)	14,513 (49.8)	3.51 (1.92)	0–13	2	3	5	7561
Older females	7088	2.08 (1.24)	0–10	1	2	3	3256
Younger females	4606	1.67 (0.91)	0–7	1	1	2	2658
Older males	7568	2.18 (1.26)	0–9	1	2	3	3320
Younger males	9907	2.61 (1.49)	0–10	1	2	3	4903
Ischemic heart diseases (I20-I25)	9075 (31.1)	2.44 (1.40)	0–10	1	2	3	5896
Cardiac arrhythmias and heart failure (I44-I50)	20,094 (68.9)	4.71 (2.53)	0–18	3	4	6	9555
Atrial fibrillation and flutter (I48)	9536 (32.7)	2.57 (1.53)	0–11	1	2	3	4426
Females	3704	1.56 (0.82)	0–6	1	2	3	1849
Males	5832	1.89 (1.10)	0–10	1	2	2	2577
Older (≥71 yr)	4227	1.65 (0.91)	0–8	1	1	2	2372
Younger (< 71 yr)	5309	1.78 (1.00)	0–7	1	1	2	2214
Older females	2315	1.33 (0.61)	0–5	1	1	2	1276
Younger females	1389	1.17 (0.43)	0–4	1	1	1	644
Older males	1912	1.29 (0.57)	0–5	1	1	1	1096
Younger males	3920	1.56 (0.83)	0–7	1	1	2	1570

*SD* Standard deviation; *yr* years, P (25): 25% percentiles, P (75): 75% percentiles

Descriptive statistics and Spearman’s correlation of traffic-related pollutants, H_2_S, and meteorological variables are presented in Table 2. For each pollutant, the mean concentration was higher in the winter months (November–April) than in the summer months (May–October), showing a seasonal pattern. NO_2_ had the highest mean (20.7 μg/m^3^) and the highest interquartile range (IQR), 15.8 μg/m^3^. SO_2_ had the lowest mean (2.51 μg/m^3^) and the lowest IQR (1.2 μg/m^3^), although the maximum value for SO_2_ was 409 μg/m^3^, as shown in Table 2. Spearman’s correlation was used to evaluate how pollution had evolved during the study period. NO_2_ did not change significantly over the study period, while PM_10_ and PM_2.5_ concentrations were reduced over the study period (Table 2). SO_2_ and H_2_S concentrations did however increase during the study period (Table 2).

**Table 2:** Descriptive statistics of 24-h concentration levels (μg/m^**3**^) of pollutants and meteorological data in the Reykjavík capital area during the study period, 2006–2017 and Spearman’s correlation coefficients between daily concentration of pollutant and calendar days through the study period

	PM_10_	PM_2,5_	NO_2_	SO_2_	H_2_S	TEMP °C	RH (%)
Mean (SD)	20.5 (19.7)	12.5 (21.8)	20.7 (15.0)	2.51 (13.8)	2.98 (5.2)	5.5 (4.9)	74.9 (10.6)
Summer^a^ mean (SD)	17.4 (14.9)	10.8 (16.2)	16.2 (9.9)	2.48 (14.1)	2.08 (3.1)	9.1 (3.2)	74.6 (9.8)
Winter^b^ mean (SD)	23.6 (23.2)	14.2 (26.1)	25.3 (17.6)	2.54 (13.5)	3.90 (6.6)	1.9 (3.4)	75.1 (11.3)
Range	2.4–381	0–423	0–119	0–409	0–96	−10.5-17.7	37–97
Median	15.1	7.0	16.6	1.1	1.2	5.6	77.0
Interquartile range	11.6	8.2	15.8	1.2	2.7	7.9	15.0
Correlation coefficients	−0.17^c^	−0.12^c^	0.023	0.11^c^	0.13^c^		

^a^May 1st to October 31st

^b^November 1st to April 30th

^c^Statistically significant correlation with *p*-value < 0.05

*SD* Standard deviation; *H*_*2*_*S* Hydrogen sulphide; *NO*_*2*_ Nitrogen dioxide; *PM*_*10*_ Particulate matter ≤10 μm in diameter; *PM*_*2.5*_ Particulate matter ≤2.5 μm in diameter; *RH* Relative humidity; *SO*_*2*_ Sulphur dioxide; *TEMP* Temperature

In the single pollutant analyses, positive associations were observed for exposure to NO_2_ at lag 0, and the increased risks of heart diseases (ICD-10 codes: I20-I25, I44-I50), cardiac arrhythmias or heart failure (ICD-10 codes: I44-I50), and AF (ICD-10 code: I48), the increased risks were OR 1.013 (95% CI 1.003–1.023), OR 1.020 (95% CI 1.008–1.032), and OR 1.023 (95% CI 1.005–1.040), respectively, per 10 μg/m^3^ increase of NO_2_, shown in Table D, Additional file 1.

**Fig. 1:**
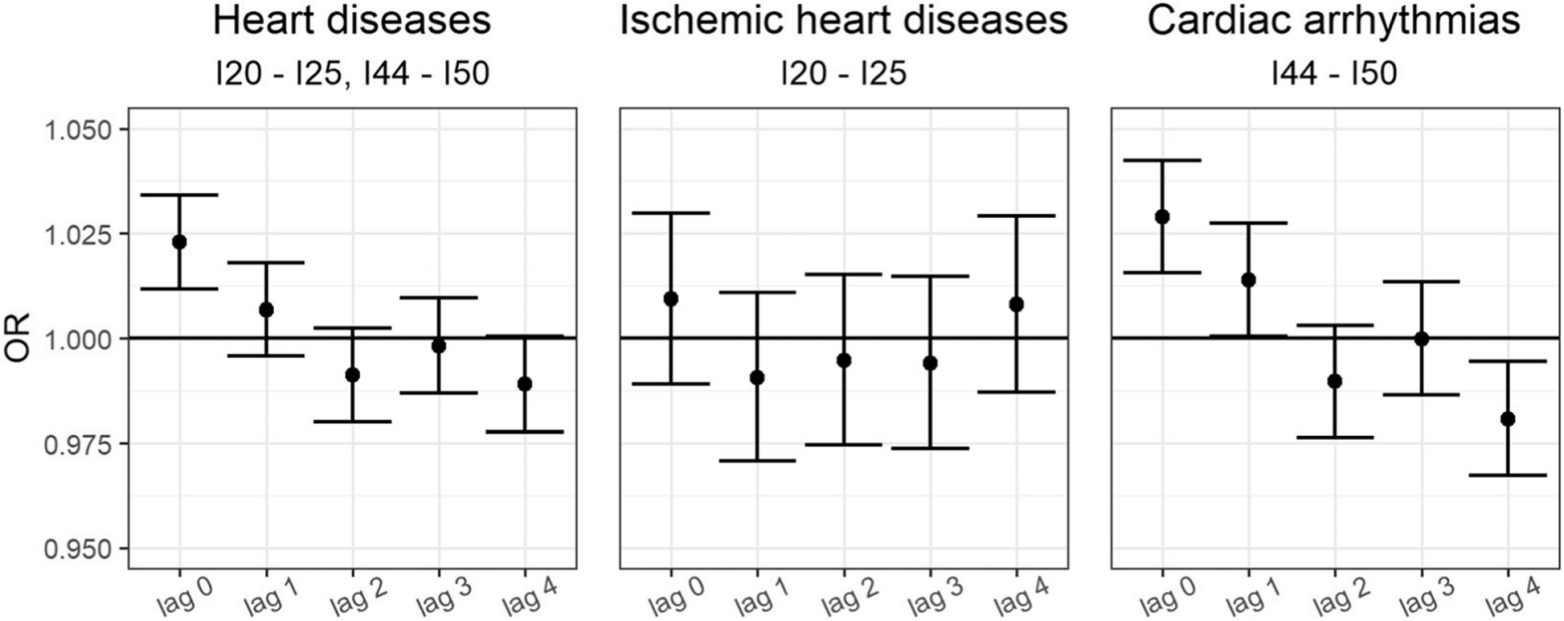
The odds ratio (OR) and bars showing 95% CI per 10 μg/m^**3**^ increase in NO_2_ concentrations and emergency hospital visits for heart diseases (ICD-10: I20-I25, I44-I50), ischemic heart diseases (ICD-10: I20-I25), and cardiac arrhythmias (ICD-10: I44-I50), at lag 0 to lag 4 of exposure

In examining the daily lag exposure to NO_2_ and unstratified emergency hospital visits for heart diseases (ICD-10: I20-I25, I44-I50), a positive association was observed for lag 0 in the multivariate model, and the increased risk of heart diseases was OR 1.023 (95% CI 1.012–1.034) per 10 μg/m^3^ increase of NO_2_ (Fig. 1, Table 3). No significant associations were shown for other pollutants and unstratified emergency hospital visits for heart diseases (Table 3), except that positive association was observed at lag 3, where the increased risk of heart diseases was OR 1.009 (95% CI 1.001–1.016) per 10 μg/m^3^ increase of PM_2.5_ (Table 3). For lag 0–1 increased risks of heart diseases, cardiac arrhythmias or heart failure, and AF were OR 1.022 (95% CI 1.008–1.036), OR 1.033 (95% CI 1.016–1.050), and OR 1.037 (95% CI 1.013–1.061), respectively, per 10 μg/m^3^ increase of NO_2_ (Table C, Additional file 1). For lag 0–2 increased risk of cardiac arrhythmias or heart failure was OR 1.023 (95% CI 1.004–1.043) per 10 μg/m^3^ increase of NO_2_ (Table C, Additional file 1).

**Table 3:** Odds ratios (OR) and 95% confidence intervals (CI) for the daily emergency hospital visits for heart diseases (ICD-10 codes: I20-I25, I44-I50) in Reykjavik capital area associated with 10 μg/m^**3**^ increase in NO_2_, PM_10_, PM_2.5_, SO_2_ and H_2_S, adjusted for each pollutant, temperature and relative humidity, unstratified and stratified by gender and age, at lag 0 to lag 4

Categories		NO_**2**_		PM_**10**_		PM_**2,5**_		SO_**2**_		H_**2**_S	
	Lag	OR	95% CI	OR	95% CI	OR	95% CI	OR	95% CI	OR	95% CI
**All**	0	1.023	1.012–1.034	0.996	0.988–1.003	0.995	0.989–1.002	1.005	0.995–1.015	0.993	0.966–1.020
	1	1.007	0.996–1.018	1.003	0.996–1.011	0.999	0.992–1.006	1.003	0.993–1.013	0.995	0.968–1.023
	2	0.991	0.980–1.002	1.001	0.993–1.008	1.004	0.998–1.011	1.006	0.996–1.016	0.990	0.963–1.018
	3	0.998	0.987–1.009	1.009	1.001–1.016	1.006	0.999–1.013	0.999	0.998–1.010	1.012	0.985–1.040
	4	0.989	0.978–1.000	1.000	0.993–1.008	1.004	0.996–1.011	0.995	0.984–1.006	1.024	0.996–1.052
**Females**	0	1.030	1.012–1.048	1.003	0.992–1.015	0.992	0.981–1.003	1.004	0.988–1.019	0.977	0.934–1.022
	1	1.024	1.006–1.042	1.001	0.989–1.013	0.998	0.987–1.008	1.005	0.990–1.020	0.980	0.938–1.024
	2	0.986	0.969–1.004	0.996	0.984–1.008	0.999	0.989–1.010	1.008	0.992–1.024	0.986	0.944–1.031
	3	1.007	0.989–1.025	1.014	1.003–1.026	0.999	0.989–1.010	1.007	0.991–1.024	1.010	0.967–1.055
	4	0.989	0.971–1.007	0.998	0.986–1.010	1.001	0.990–1.012	0.999	0.983–1.017	1.026	0.981–1.073
**Males**	0	1.019	1.004–1.033	0.990	0.980–1.000	0.998	0.989–1.007	1.006	0.993–1.020	1.002	0.968–1.038
	1	0.996	0.982–1.010	1.005	0.996–1.014	1.000	0.991–1.009	1.001	0.989–1.014	1.005	0.971–1.041
	2	0.994	0.980–1.009	1.004	0.995–1.014	1.008	0.999–1.016	1.005	0.992–1.018	0.993	0.958–1.029
	3	0.992	0.978–1.007	1.005	0.995–1.014	1.010	1.001–1.019	0.994	0.979–1.008	1.013	0.978–1.049
	4	0.989	0.974–1.004	1.001	0.992–1.011	1.005	0.996–1.015	0.992	0.978–1.007	1.022	0.987–1.059
**Older (≥71)**	0	1.031	1.015–1.047	0.991	0.980–1.002	0.997	0.987–1.007	1.007	0.994–1.021	0.964	0.926–1.003
	1	1.017	1.002–1.034	1.003	0.993–1.014	0.996	0.986–1.006	0.999	0.985–1.013	0.971	0.933–1.010
	2	0.992	0.976–1.008	1.001	0.991–1.012	1.001	0.992–1.011	1.008	0.995–1.022	0.994	0.955–1.035
	3	1.001	0.986–1.017	1.005	0.995–1.016	1.006	0.996–1.015	0.999	0.984–1.014	1.024	0.984–1.064
	4	0.984	0.968–1.000	0.998	0.988–1.009	0.995	0.985–1.006	0.997	0.982–1.012	1.045	1.006–1.085
**Younger (< 71)**	0	1.015	0.999–1.031	1.001	0.990–1.011	0.994	0.985–1.004	1.003	0.988–1.018	1.019	0.982–1.058
	1	0.996	0.981–1.012	1.003	0.993–1.014	1.002	0.993–1.011	1.007	0.993–1.022	1.019	0.981–1.058
	2	0.991	0.975–1.007	1.000	0.989–1.011	1.007	0.998–1.017	1.004	0.989–1.019	0.986	0.949–1.025
	3	0.995	0.979–1.011	1.012	1.001–1.022	1.006	0.994–1.016	0.999	0.984–1.015	1.001	0.963–1.040
	4	0.994	0.978–1.010	1.001	0.991–1.012	1.011	1.001–1.020	0.993	0.977–1.010	1.002	0.962–1.042
**Older females**	0	1.031	1.007–1.055	0.998	0.983–1.013	0.984	0.969–0.998	1.009	0.991–1.028	0.936	0.881–0.994
	1	1.024	1.001–1.047	1.002	0.986–1.018	0.994	0.980–1.008	1.005	0.987–1.023	0.954	0.900–1.010
	2	0.986	0.964–1.009	0.999	0.985–1.015	0.999	0.985–1.012	1.013	0.994–1.033	0.965	0.910–1.024
	3	1.010	0.987–1.034	1.008	0.993–1.023	1.000	0.987–1.014	1.017	0.997–1.038	1.002	0.947–1.060
	4	0.987	0.963–1.010	0.996	0.981–1.012	0.989	0.975–1.004	1.003	0.983–1.024	1.022	0.965–1.082
**Younger females**	0	1.030	1.002–1.059	1.010	0.993–1.028	1.002	0.986–1.018	0.993	0.966–1.021	1.035	0.968–1.016
	1	1.024	0.996–1.053	1.000	0.982–1.019	1.002	0.987–1.018	1.005	0.989–1.033	1.019	0.953–1.090
	2	0.987	0.959–1.016	0.990	0.970–1.010	0.999	0.983–1.017	0.997	0.968–1.026	1.014	0.949–1.084
	3	1.003	0.974–1.032	1.023	1.005–1.041	0.999	0.982–1.016	0.988	0.958–1.019	1.023	0.955–1.094
	4	0.993	0.964–1.022	1.000	0.982–1.019	1.017	0.999–1.034	0.993	0.964–1.023	1.032	0.961–1.108
**Older males**	0	1.032	1.010–1.054	0.984	0.969–0.999	1.008	0.995–1.021	1.005	0.985–1.025	0.987	0.935–1.041
	1	1.012	0.991–1.034	1.005	0.990–1.019	0.998	0.984–1.012	0.991	0.970–1.013	9.987	0.935–1.042
	2	0.997	0.975–1.019	1.003	0.989–1.017	1.004	0.990–1.017	1.003	0.985–1.022	1.021	0.967–1.078
	3	0.994	0.972–1.016	1.003	0.988–1.018	1.011	0.997–1.026	0.978	0.955–1.003	1.044	0.989–1.102
	4	0.982	0.960–1.004	1.001	0.986–1.016	1.002	0.988–1.017	0.991	0.970–1.013	1.063	1.010–1.119
**Younger males**	0	1.009	0.990–1.028	0.995	0.982–1.008	0.990	0.978–1.002	1.007	0.989–1.026	1.013	0.969–1.060
	1	0.983	0.965–1.002	1.005	0.993–1.017	1.002	0.990–1.014	1.008	0.991–1.025	1.019	0.973–1.067
	2	0.993	0.973–1.012	1.005	0.992–1.018	1.010	0.999–1.022	1.006	0.989–1.024	0.973	0.928–1.020
	3	0.991	0.972–1.011	1.006	0.993–1.019	1.009	0.998–1.021	1.004	0.986–1.023	0.990	0.945–1.038
	4	0.995	0.975–1.015	1.002	0.989–1.015	1.008	0.996–1.020	0.994	0.974–1.013	0.988	0.941–1.037

In the stratified analysis for heart diseases, females and those aged 71 years or older had higher effect estimates for the association between NO_2_ exposure and heart diseases. For females, increased risks were OR 1.030 (95% CI 1.012–1.048) at lag 0, and OR 1.024 (95% CI 1.006–1.042) at lag 1, per 10 μg/m^3^ increase of NO_2_. For those aged 71 years or older, the increased risk was OR 1.031 (95% CI 1.015–1.047) at lag 0, and OR 1.017 (95% CI 1.002–1.034) at lag 1 (Table 3). Among older females and younger females, NO_2_ exposure had similar effect estimates as for all females (Table 3). Looking at the effect estimates for the association between NO_2_ exposure and heart diseases among males, the increased risk was OR 1.019 (95% CI 1.004–1.033) at lag 0, per 10 μg/m^3^ increase of NO_2_; and for older males the increased risk was OR 1.032 (95% CI 1.010–1.054) at lag 0 (Table 3). In this analysis a positive association was observed among females at lag 3, where the increased risk of heart diseases was OR 1.014 (95% CI 1.003–1.026), per 10 μg/m^3^ increase of PM_2.5_. Among those younger than 71 years at lag 3, the increased risk of heart diseases was OR 1.012 (95% CI 1.001–1.022) per 10 μg/m^3^ increase of PM_2.5_, and among females younger than 71 years at lag 3, the increased risk of heart diseases was OR 1.023 (95% CI 1.005–1.043) per 10 μg/m^3^ increase of PM_2.5_, (Table 3).

In the analysis of the association between daily lag exposure to the pollutants in the study and unstratified emergency hospital visits for ischemic heart diseases (ICD-10: I20-I25) no significant association was observed at any lag in the multivariate model, (Fig. 1, Table A, Additional file 1). The association between daily lag exposure to NO_2_ and unstratified emergency hospital visits for cardiac arrhythmias/heart failure (ICD-10: I44-I50) showed a positive association at lag 0 and lag 1 in the multivariate model, the increased risk of cardiac arrhythmias/heart failure were OR 1.029 (95% CI 1.016–1.042) at lag 0, and OR 1.014 (95% CI 1.00–1.027) at lag 1, per 10 μg/m^3^ increase of NO_2_ (Fig. 1, Table B, Additional file 1). In this analysis a positive association was also observed for cardiac arrhythmias/heart failure at lag 3, OR 1.013 (95% CI 1.004–1.022) per 10 μg/m^3^ increase of PM_2.5_, and OR 1.010 (95% CI 1.001–1.018) per 10 μg/m^3^ increase of PM_10_ (Table B, Additional file 1).

**Fig. 2:**
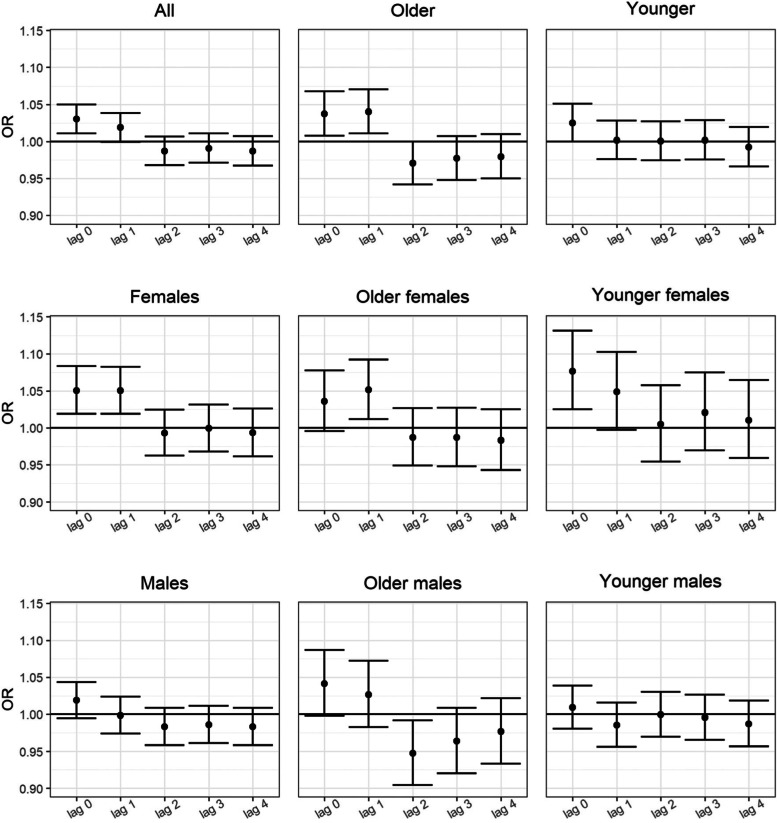
The odds ratio (OR) and bars showing 95% CI of atrial fibrillation and flutter (ICD-10 code I48) per 10 μg/m3 increase in NO2 concentrations in multiple-pollutant models at lag 0 to lag 4 for unstratified material and different strata

For the association between daily lag exposure to NO_2_ and unstratified emergency hospital visits for AF (ICD-10: I48) a positive association was observed for lag 0 in the multivariate model; the increased risk of AF was OR 1.030 (95% CI 1.011–1.049) per 10 μg/m^3^ increase of NO_2_ (Fig. 2, Table 4). No significant associations were shown for other pollutants and unstratified emergency hospital visits for AF (Fig. 2, Table 4).

**Table 4:** Odds ratios (OR) and 95% confidence intervals (CI) for the daily emergency hospital visits for atrial fibrillation and flutter (ICD-10 code: I48) in Reykjavik capital area associated with 10 μg/m^**3**^ increase in NO_2_, PM_10_, PM_2.5_, SO_2_ and H_2_S, adjusted for each pollutant, temperature and relative humidity, unstratified and stratified by gender and age, at lag 0 to lag 4

Categories		NO_**2**_		PM_**10**_		PM_**2,5**_		SO_**2**_		H_**2**_S	
	Lag	OR	95% CI	OR	95% CI	OR	95% CI	OR	95% CI	OR	95% CI
**All**	0	1.030	1.011–1.049	0.989	0.976–1.004	0.986	0.974–0.999	1.006	0.989–1.023	0.972	0.925–1.021
	1	1.019	0.999–1.038	0.999	0.987–1.013	0.992	0.980–1.005	1.006	0.991–1.022	0.992	0.945–1.041
	2	0.987	0.968–1.007	1.004	0.989–1.017	1.003	0.990–1.015	1.003	0.986–1.019	1.009	0.961–1.060
	3	0.991	0.971–1.011	1.009	0.996–1.023	1.001	0.988–1.014	1.006	0.989–1.024	1.024	0.975–1.075
	4	0.987	0.967–1.007	0.999	0.986–1.013	1.002	0.989–1.016	1.000	0.984–1.017	1.039	0.989–1.091
**Females**	0	1.051	1.019–1.083	1.000	0.979–1.022	0.990	0.971–1.009	0.998	0.971–1.025	0.936	0.863–1.015
	1	1.050	1.019–1.083	0.997	0.975–1.019	0.994	0.974–1.013	1.009	0.988–1.031	0.976	0.906–1.051
	2	0.993	0.962–1.024	1.009	0.988–1.032	0.996	0.976–1.018	1.014	0.988–1.041	0.979	0.904–1.059
	3	0.999	0.968–1.031	1.029	1.007–1.052	0.995	0.974–1.016	1.012	0.987–1.038	1.034	0.958–1.116
	4	0.993	0.962–1.026	1.011	0.990–1.032	1.002	0.981–1.023	0.997	0.972–1.024	1.019	0.942–1.102
**Males**	0	1.019	0.995–1.043	0.,983	0.965–1.000	0.984	0.968–0.999	1.012	0.991–1.034	0.993	0.933–1.057
	1	0.999	0.974–1.024	1.002	0.985–1.018	0.991	0.975–1.007	1.004	0.981–1.027	1.001	0.939–1.068
	2	0.983	0.958–1.008	0.999	0.982–1.017	1.006	0.991–1.022	0.994	0.971–1.017	1.029	0.967–1.095
	3	0.986	0.961–1.011	0.997	0.980–1.016	1.005	0.989–1.022	1.002	0.978–1.026	1.017	0.954–1.083
	4	0.983	0.958–1.009	0.991	0.973–1.009	1.003	0.986–1.020	1.003	0.981–1.025	1.053	0.989–1.121
**Older (≥71)**	0	1.037	1.008–1068	0.988	0.968–1.008	0.990	0.971–1.009	1.003	0.980–1.027	0.922	0.852–0.998
	1	1.040	1.011–1.071	0.994	0.975–1.014	0.987	0.968–1.006	0.999	0.977–1.022	0.937	0.871–1.008
	2	0.970	0.942–0.999	1.005	0.985–1.025	0.997	0.977–1.016	1.010	0.989–1.032	0.985	0.913–1.064
	3	0.977	0.948–1.007	1.008	0.988–1.030	1.002	0.982–1.022	1.017	0.992–1.042	1.007	0.936–1.083
	4	0.979	0.950–1.010	1.007	0.987–1.028	0.995	0.974–1.016	1.009	0.979–1.023	1.036	0.966–1.111
**Younger (< 71)**	0	1.025	0.999–1.051	0.992	0.973–1.010	0.984	0.967–1.000	1.010	0.986–1.034	1.007	0.945–1.072
	1	1.002	0.976–1.028	1.004	0.986–1.022	0.997	0.980–1.013	1.013	0.991–1.035	1.039	0.974–1.110
	2	1.000	0.974–1.027	1.002	0.983–1.021	1.007	0.991–1.024	0.990	0.962–1.020	1.026	0.962–1.094
	3	1.001	0.975–1.028	1.011	0.992–1.030	1.001	0.984–1.018	0.997	0.972–1.022	1.038	0.972–1.108
	4	0.992	0.966–1.019	0.993	0.975–1.012	1.008	0.990–1.025	1.001	0.975–1.027	1.041	0.972–1.115
**Older females**	0	1.036	0.996–1.077	0.997	0.971–1.024	0.985	0.959–1.011	1.001	0.968–1.035	0.840	0.749–0.941
	1	1.051	1.012–1.092	0.990	0.963–1.017	0.997	0.973–1.021	1.007	0.980–1.035	0.925	0.841–1.018
	2	0.987	0.949–1.027	1.019	0.991–1.047	1.003	0.977–1.031	1.022	0.993–1.051	0.936	0.843–1.040
	3	0.987	0.948–1.027	1.037	1.009–1.066	0.999	0.974–1.026	1.035	1.004–1.067	0.997	0.902–1.101
	4	0.983	0.943–1.025	1.022	0.996–1.048	0.983	0.954–1.012	0.998	0.969–1.029	1.029	0.935–1.133
**Younger females**	0	1.077	1.025–1.131	1.006	0.970–1.044	0.998	0.969–1.029	0.994	0.949–1.043	1.079	0.958–1.215
	1	1.049	0.997–1.102	1.009	0.974–1.046	0.988	0.957–1.021	1.012	0.977–1.047	1.075	0.950–1.216
	2	1.005	0.954–1.057	0.993	0.957–1.030	0.984	0.950–1.019	0.986	0.927–1.049	1.047	0.925–1.184
	3	1.021	0.969–1.075	1.015	0.979–1.053	0.988	0.955–1.022	0.961	0.906–1.021	1.087	0.965–1.224
	4	1.010	0.959–1.064	0.988	0.952–1.026	1.025	0.994–1.057	0.995	0.944–1.049	0.997	0.871–1.141
**Older males**	0	1.041	0.998–1.087	0.975	0.944–1.007	0.997	0.969–1.025	1.006	0.972–1.041	1.028	0.918–1.151
	1	1.026	0.983–1.072	0.999	0.971–1.028	0.972	0.942–1.003	0.986	0.947–1.026	0.954	0.850–1.070
	2	0.947	0.904–0.992	0.990	0.961–1.019	0.989	0.961–1.018	0.997	0.965–1.029	1.054	0.943–1.178
	3	0.963	0.920–1.009	0.972	0.939–1.005	1.004	0.973–1.037	0.987	0.945–1.031	1.013	0.909–1.130
	4	0.977	0.933–1.022	0.984	0.950–1.018	1.008	0.978–1.039	1.004	0.973–1.036	1.045	0.943–1.157
**Younger males**	0	1.009	0.980–1.038	0.987	0.965–1.008	0.978	0.958–0.997	1.016	0.988–1.045	0.979	0.908–1.055
	1	0.985	0.956–1.016	1.002	0.982–1.023	0.999	0.981–1.019	1.013	0.985–1.041	1.024	0.948–1.106
	2	0.999	0.969–1.030	1.005	0.983–1.027	1.014	0.996–1.033	0.991	0.959–1.025	1.020	0.946–1.099
	3	0.996	0.965–1.027	1.009	0.988–1.031	1.005	0.986–1.025	1.010	0.981–1.039	1.017	0.940–1.100
	4	0.987	0.957–1.018	0.995	0.974–1.016	1.001	0.980–1.022	1.003	0.973–1.033	1.057	0.976–1.145

In the stratified analysis for AF, females and those aged 71 years or older had higher effect estimates for the association between NO_2_ exposure and AF: for females the increased risk was OR 1.051 (95% CI 1.019–1.083) at lag 0, and OR 1.050 (95% CI 1.019–1.083) at lag 1, per 10 μg/m^3^ increase of NO_2_; and for those aged 71 years or older the increased risk was OR 1.037 (95% CI 1.008–1.068) at lag 0, and OR 1.040 (95% CI 1.011–1.071) at lag 1 (Fig. 2, Table 4). The only significant association shown for other pollutants and emergency hospital visits for AF was the positive association observed at lag 3, where the increased risk of AF was OR 1.029 (95% CI 1.007–1.052) per 10 μg/m^3^ increase of PM_2.5_ (Table 4). Among older females and younger females, the NO_2_ exposure had similar effect estimates at lag 0 and lag 1 as for all females (Table 4). In this analysis a positive association was observed among older females at lag 3, where the increased risk of AF was OR 1.037 (95% CI 1.009–1.066) per 10 μg/m^3^ increase of PM_2.5_. However, although in this analysis of AF, elevated effect estimates for the association between NO_2_ exposure and AF at lag 0 were observed for older males and males 70 years and younger, none of these were statistically significant (Table 4).

Sensitive analysis of the association between daily lag exposure to NO_2_ and emergency hospital visits for AF (ICD-10 codes I48) when restricting the calculation to ED visits only did not change the main results substantially.

## Discussion

The main results of this study were the significant association between increased NO_2_ concentrations and emergency hospital visits for heart diseases (ICD-10 code I20-I25, I44-I50) at lag 0. The association was strongest among patients diagnosed with AF (ICD-10 code I48) at lag 0 and lag 1, and it seemed that females and particularly younger females were more susceptible to NO_2_ exposure. Concerning other pollutants, the association between the exposure and heart diseases or AF did not show a pattern similar to the NO_2_ increase. Supporting this are the findings from the single pollutant analyses, as well as the findings from the lag 0–1 analyses.

Some previous studies have found a positive association between NO_2_ levels and cases of AF [5, 7–9]. The results of the present study indicate that the association between NO_2_ and emergency admission for AF at lag 0 is consistent with the results of the recent multilocation study which found an association between NO_2_ and cardiovascular mortality most prominently at lag 1, but not at lag 0 [12]. The association between AF and long-term exposure to NO_2_ has also been observed in population-based studies [25, 26]. Ambient NO_2_ concentrations have also been found to be associated with cardiac repolarization abnormalities in healthy adults [27]. However, other studies have not found association between exposure to air pollution and AF onset [10, 11], while a study on patients with cardiac implantable electronic devices showed an association between increase in particulate matter and AF, but no association was noted with NO_2_ [28].

It is a strength of this study that it is population-based since the hospital data was obtained from the only acute care hospital serving the population of the Reykjavik capital area, the LUH. Another strength is the time-stratified case-crossover design of the study, which excludes the confounding of individual characteristics and adjusts for time trends such as weekdays and seasons. Yet another strength of the present study is that data of emergency hospital visits were collected prospectively from the population-based hospital register, virtually excluding the risk of information bias from individuals knowing their exposure status. Furthermore, the main result of this study, namely the strong associations between NO_2_ exposure and younger females (< 71 years) diagnosed with AF (ICD-10 code I48), is a strength because younger individuals have fewer diseases on average, and therefore this association is less likely to be confounded by other diseases. It is also noteworthy that visits of cases of AF per day are evenly distributed over the study period and are on average one or two cases per day, thus limiting the risk of overlapping the sets of case and control days.

There were a few limitations to this study. First, the data on pollution was from one measurement station in Reykjavik capital area at GRE and did not contain data on individual exposures. However, to test whether the measurements from GRE were indicative for the whole capital area, correlation calculations were done between the concentrations at GRE and concentrations from another measurement station located in Kopavogur, one of the municipalities included in the capital area, covering three years of the study period. The correlation coefficient was high for NO_2_ (0.78), H_2_S (0.84), and SO_2_ (0.98), but the coefficient for PM_10_ was lowest (0.44). However, these coefficients indicate that measurements at GRE describe the situation with reasonable accuracy for the whole population in the study area.

Another limitation is that only the primary discharge diagnosis was included in the study. The patients may have other important underlying diseases which could modify the results. Further, the quality of the routine medical diagnoses at the LUH has not been evaluated in a separate study, a weakness the study shares with other studies relying on hospital records.

The third limitation was that the main results were found at lag 0, which means that it may not be clear whether the emergency hospital visits occurred after the pollution increased because both pollution data and hospital data were calculated on daily basis (not hourly basis). However, the ORs were significantly increased at lag 1 for AF among females, older age group, and older females, and the OR was also increased at lag 0–1, thus indicating that increased exposure occurred before the visits. The present finding of AF, a certain cardiac diagnosis, at lag 0 is consistent with increased cardiovascular mortality at lag 1, found in another study [12].

The fourth limitation is that visits may be double-counted; although readmissions within 10 days with the same primary discharge diagnosis were excluded, it is still possible that some people first went to the ED and were subsequently admitted into a hospital ward under a different diagnosis than they got at the ED. To test whether this may distort the main results of the association between increased NO_2_ and emergency hospital visits, the data was restricted to ED visits only. These calculations revealed similar estimates for the association between NO_2_ and all heart diseases in the unstratified model as well as when the restriction was made to AF. Double counting of patients due to different diagnosis at the ED and in the hospital wards is thus unlikely to be distorting the results.

The fifth limitation is that the study population consisted of those aged 18 years and older, limiting the generalisability of the results to younger age groups.

The sixth limitation is that because of the diurnal distribution of the admissions and visits to LUH, it was not realistic to achieve a narrower time frame than 24 h in the association analysis.

The seventh limitation is the possibility that pollution from the volcanic eruption of the Eyjafjallajökull in 2010 and the Holuhraun eruption in 2014 to 2015 may have confounded the results. The Eyjafjallajökull eruption was only found to affect the local population living near the volcano, not the population in Reykjavik capital area, and no serious health problems were found [29]. On the contrary, short term exposure to SO_2_ and exposure to mature volcanic plume originated from the Holuhraun eruption, occurring 250 km from Reykjavik, was associated with an increase in dispensing of asthma medication and increase in health care utilisation for respiratory diseases in the Reykjavik capital area for 4 months in the year 2014 [30, 31]. Whether this volcanic emission has affected the short or long-term cardiovascular-related health of the population of the Reykjavik capital area is unknown.

The eighth limitation of this study is related to the outcome measures, the hospital data did not contain information on the exact onset of the diseases under study, or whether the patients had had an exacerbation of symptoms in relation to their attendance to the hospital.

There were several stratifications and restrictions used to explore the possible association between air pollutants and emergency hospital visits in this study. This may give rise to concerns about multiple comparisons; however, it has been stated that no adjustments are needed [32–34]. To deal with multiple comparisons, it has been argued that clearly describing what tests of significance were performed, and for what purpose they were done, is the best way to address this phenomenon [33].

## Conclusions

The results indicate a positive association between short-term increase in NO_2_ concentrations and emergency hospital visits for heart diseases in the Reykjavik capital area, especially visits for atrial fibrillation and flutter, and particularly among females. This is the first study in Reykjavik, Iceland, that finds an association between air pollution and cardiac arrhythmias. Furthermore, this is the first study in Iceland where the possible effects of PM_2.5_ on health indicators are evaluated, but even though significant associations were now and then found at lag 3 between PM_2.5_ and emergency hospital visits for heart diseases and AF, the associations were weak and did not show a consistent pattern after restrictions were placed on the disease categories. Same was the case for PM_10_ and SO_2_: there were some significant associations found between those pollutants and emergency hospital visits, but not with a clear pattern like the association between NO_2_ and hospital visits.

## Supplementary Information

**Additional file 1:** **Table A.** Odds ratios (OR) and 95% confidence intervals (CI) for the daily emergency hospital visits for ischemic heart diseases (ICD-10 codes: I20-I25) in Reykjavik capital area associated with 10 μg/m^3^ increase in NO_2_, PM_10_, PM_2.5_, SO_2_ and H_2_S, adjusted for each pollutant, temperature and relative humidity, at lag 0 to lag 4. **Table B.** Odds ratios (OR) and 95% confidence intervals (CI) for the daily emergency hospital visits for cardiac arrhythmias or heart failure (ICD-10 codes: I44-I50) in Reykjavik capital area associated with 10 μg/m^3^ increase in NO_2_, PM_10_, PM_2.5_, SO_2_ and H_2_S, adjusted for each pollutant, temperature and relative humidity, at lag 0 to lag 4. **Table C.** Odds ratios (OR) and 95% confidence intervals (CI) for the daily emergency hospital visits for heart diseases (ICD-10 codes: I20-I25, I44-I50; I20-I25; I44-I50; and I48) in Reykjavik capital area associated with 10 μg/m^3^ increase in NO_2_, PM_10_, PM_2.5_, SO_2_ and H_2_S, adjusted for each pollutant, temperature and relative humidity, at lag 0–1 (moving average of lags 0, and 1) and at lag 0–2 (moving average of lags 0, 1, and 2). **Table D.** Odds ratios (OR) and 95% confidence intervals (CI) for the daily emergency hospital visits for heart diseases (ICD-10 codes: I20-I25, I44-I50; I20-I25; I44-I50; and I48) in Reykjavik capital area associated with 10 μg/m^3^ increase in NO_2_, PM_10_, PM_2.5_, SO_2_ and H_2_S, in single pollutant models, at lag 0 to lag 4.

## Data Availability

The hospital data contain sensitive individual-level information which is not publicly available. It can be made available to researchers after obtaining approval of a formal application to the National Bioethics Committee and the Scientific Committee of LUH. The dataset of air pollution used and analysed during the current study are available from the corresponding author on reasonable request.

## Abbreviations

μm: Micrometre
AF: Atrial fibrillation
CI: Confidence interval
CVD: Cardiovascular diseases
ED: Emergency department
GRE: Air quality measurement station located at Grensasvegur-Miklabraut intersection in Reykjavik
H_2_S: Hydrogen sulphide
ICD-10: International Classification of Diseases 10th edition
IQR: Interquartile range
IHD: Ischemic heart disease
km: Kilometre
LUH: Landspitali University Hospital
NO_2_: Nitrogen dioxide
OR: Odds ratio
PM_10_: Particulate matter less than 10 μm in aerodynamic diameter
PM_2.5_: Particulate matter less than 2.5 μm in aerodynamic diameter
RH: Relative humidity
SAGA: Register of Hospital-treated Patients in Iceland
SO_2_: Sulphur dioxide
yr: Years
