# Supplementary material for: Ambient nitrogen dioxide is associated with emergency hospital visits for atrial fibrillation: a population-based case-crossover study in Reykjavik, Iceland
Source: Environ Health. 2022 Jan 3;21:2. doi: 10.1186/s12940-021-00817-9 (PMC8722049; doi:10.1186/s12940-021-00817-9)
Supplement: Supplementary file 1 — Additional file 1: Table A. Odds ratios (OR) and 95% confidence intervals (CI) for the daily emergency hospital visits for ischemic heart diseases (ICD-10 codes: I20-I25) in Reykjavik capital area associated with 10 μg/m3 increase in NO2, PM10, PM2.5, SO2 and H2S, adjusted for each pollutant, temperature and relative humidity, at lag 0 to lag 4. Table B. Odds ratios (OR) and 95% confidence intervals (CI) for the daily emergency hospital visits for cardiac arrhythmias or heart failure (ICD-10 codes: I44-I50) in Reykjavik capital area associated with 10 μg/m3 increase in NO2, PM10, PM2.5, SO2 and H2S, adjusted for each pollutant, temperature and relative humidity, at lag 0 to lag 4. Table C. Odds ratios (OR) and 95% confidence intervals (CI) for the daily emergency hospital visits for heart diseases (ICD-10 codes: I20-I25, I44-I50; I20-I25; I44-I50; and I48) in Reykjavik capital area associated with 10 μg/m3 increase in NO2, PM10, PM2.5, SO2 and H2S, adjusted for each pollutant, temperature and relative humidity, at lag 0–1 (moving average of lags 0, and 1) and at lag 0–2 (moving average of lags 0, 1, and 2). Table D. Odds ratios (OR) and 95% confidence intervals (CI) for the daily emergency hospital visits for heart diseases (ICD-10 codes: I20-I25, I44-I50; I20-I25; I44-I50; and I48) in Reykjavik capital area associated with 10 μg/m3 increase in NO2, PM10, PM2.5, SO2 and H2S, in single pollutant models, at lag 0 to lag 4. [file 12940_2021_817_MOESM1_ESM.docx]

# Appendix

Table A. Odds ratios (OR) and 95% confidence intervals (CI) for the daily emergency hospital visits for ischemic heart diseases (ICD-10 codes: I20-I25) in Reykjavik capital area associated with 10 µg/m³ increase in NO_2_, PM_10_, PM_2.5_, SO_2_ and H_2_S, adjusted for each pollutant, temperature and relative humidity, at lag 0 to lag 4.

|  |  | **NO_2_** | | **PM_10_** | | **PM_2,5_** | | **SO_2_** | | **H_2_S** | |
| --- | --- | --- | --- | --- | --- | --- | --- | --- | --- | --- | --- |
| **ICD-10 codes** | **Lag** | **OR** | **95% CI** | **OR** | **95% CI** | **OR** | **95% CI** | **OR** | **95% CI** | **OR** | **95% CI** |
| **I20 – I25** | 0 | 1.009 | 0.989-1.030 | 0.984 | 0.970-0.998 | 0.998 | 0.986-1.010 | 1.002 | 0.983-1.022 | 1.039 | 0.993-1.087 |
|  | 1 | 0.991 | 0.971-1.012 | 0.997 | 0.984-1.010 | 0.999 | 0.987-1.011 | 1.004 | 0.986-1.024 | 1.013 | 0.966-1.061 |
|  | 2 | 0.995 | 0.975-1.015 | 1.001 | 0.989-1.014 | 0.999 | 0.988-1.011 | 1.009 | 0.992-1.026 | 1.007 | 0.961-1.055 |
|  | 3 | 0.994 | 0.974-1.015 | 0.999 | 0.986-1.012 | 0.998 | 0.986-1.011 | 0.994 | 0.975-1.013 | 1.024 | 0.977-1.074 |
|  | 4 | 1.008 | 0.987-1.029 | 0.999 | 0.986-1.013 | 0.997 | 0.985-1.010 | 0.990 | 0.969-1.011 | 0.996 | 0.948-1.046 |

Table B. Odds ratios (OR) and 95% confidence intervals (CI) for the daily emergency hospital visits for cardiac arrhythmias or heart failure (ICD-10 codes: I44-I50) in Reykjavik capital area associated with 10 µg/m³ increase in NO_2_, PM_10_, PM_2.5_, SO_2_ and H_2_S, adjusted for each pollutant, temperature and relative humidity, at lag 0 to lag 4.

|  |  | **NO_2_** | | **PM_10_** | | **PM_2,5_** | | **SO_2_** | | **H_2_S** | |
| --- | --- | --- | --- | --- | --- | --- | --- | --- | --- | --- | --- |
| **ICD-10 codes** | **Lag** | **OR** | **95% CI** | **OR** | **95% CI** | **OR** | **95% CI** | **OR** | **95% CI** | **OR** | **95% CI** |
| **I44 – I50** | 0 | 1.029 | 1.016-1.042 | 1.001 | 0.992-1.010 | 0.994 | 0.986-1.003 | 1.006 | 0.995-1.019 | 0.969 | 0.936-1.003 |
|  | 1 | 1.014 | 1.000-1.027 | 1.006 | 0.998-1.015 | 0.999 | 0.991-1.007 | 1.002 | 0.991-1.014 | 0.987 | 0.954-1.021 |
|  | 2 | 0.990 | 0.976-1.003 | 1.000 | 0.991-1.010 | 1.007 | 0.999-1.015 | 1.005 | 0.992-1.017 | 0.982 | 0.949-1.016 |
|  | 3 | 0.999 | 0.986-1.013 | 1.013 | 1.004-1.022 | 1.010 | 1.001-1.018 | 1.002 | 0.989-1.015 | 1.006 | 0.972-1.040 |
|  | 4 | 0.981 | 0.967-0.994 | 1.000 | 0.991-1.009 | 1.006 | 0.998-1.015 | 0.998 | 0.985-1.011 | 1.037 | 1.003-1.072 |

Table C. Odds ratios (OR) and 95% confidence intervals (CI) for the daily emergency hospital visits for heart diseases (ICD-10 codes: I20-I25, I44-I50; I20-I25; I44-I50; and I48) in Reykjavik capital area associated with 10 µg/m³ increase in NO_2_, PM_10_, PM_2.5_, SO_2_ and H_2_S, adjusted for each pollutant, temperature and relative humidity, at lag 0-1 (moving average of lags 0, and 1) and at lag 0-2 (moving average of lags 0, 1, and 2).

|  |  | **NO_2_** | | **PM_10_** | | **PM_2,5_** | | **SO_2_** | | **H_2_S** | |
| --- | --- | --- | --- | --- | --- | --- | --- | --- | --- | --- | --- |
| **ICD-10 codes** | **Lag** | **OR** | **95% CI** | **OR** | **95% CI** | **OR** | **95% CI** | **OR** | **95% CI** | **OR** | **95% CI** |
| **I20 – I25, I44 – I50** | 0-1 | 1.022 | 1.008-1.036 | 0.999 | 0.990-1.009 | 0.997 | 0.989-1.005 | 1.006 | 0.993-1.019 | 0.986 | 0.953-1.020 |
|  | 0-2 | 1.013 | 0.998-1.029 | 1.000 | 0.989-1.011 | 1.000 | 0.991-1.009 | 1.010 | 0.995-1.026 | 0.980 | 0.943-1.020 |
| **I20 – I25** | 0-1 | 0.998 | 0.973-1.023 | 0.986 | 0.969-1.003 | 0.998 | 0.985-1.012 | 1.005 | 0.980-1.030 | 1.037 | 0.981-1.097 |
|  | 0-2 | 0.991 | 0.963-1.020 | 0.988 | 0.969-1.008 | 0.999 | 0.984-1.014 | 1.013 | 0.984-1.042 | 1.040 | 0.974-1.110 |
| **I44 – I50** | 0-1 | 1.033 | 1.016-1.050 | 1.006 | 0.994-1.017 | 0.996 | 0.986-1.005 | 1.007 | 0.991-1.022 | 0.960 | 0.920-1.001 |
|  | 0-2 | 1.023 | 1.004-1.043 | 1.005 | 0.992-1.018 | 1.001 | 0.990-1.011 | 1.009 | 0.991-1.028 | 0.951 | 0.905-0.998 |
| **I48** | 0-1 | 1.037 | 1.013-1.061 | 0.992 | 0.976-1.009 | 0.986 | 0.972-1.000 | 1.010 | 0.989-1.032 | 0.966 | 0.909-1.026 |
|  | 0-2 | 1.024 | 0.996-1.052 | 0.995 | 0.976-1.015 | 0.989 | 0.973-1.005 | 1.011 | 0.986-1.037 | 0.974 | 0.909-1.045 |

Table D. Odds ratios (OR) and 95% confidence intervals (CI) for the daily emergency hospital visits for heart diseases (ICD-10 codes: I20-I25, I44-I50; I20-I25; I44-I50; and I48) in Reykjavik capital area associated with 10 µg/m³ increase in NO_2_, PM_10_, PM_2.5_, SO_2_ and H_2_S, in single pollutant models, at lag 0 to lag 4.

|  |  | **NO_2_** | | **PM_10_** | | **PM_2,5_** | | **SO_2_** | | **H_2_S** | |
| --- | --- | --- | --- | --- | --- | --- | --- | --- | --- | --- | --- |
| **ICD-10 codes** | **Lag** | **OR** | **95% CI** | **OR** | **95% CI** | **OR** | **95% CI** | **OR** | **95% CI** | **OR** | **95% CI** |
| **I20 – I25, I44 – I50** | 0 | 1.013 | 1.003-1.023 | 0.996 | 0.989-1.004 | 0.994 | 0.987-1.001 | 1.007 | 0.997-1.017 | 0.999 | 0.973-1.025 |
|  | 1 | 1.001 | 0.991-1.011 | 1.004 | 0.997-1.011 | 0.998 | 0.992-1.005 | 1.003 | 0.993-1.013 | 0.994 | 0.968-1.020 |
|  | 2 | 0.988 | 0.978-0.998 | 1.001 | 0.994-1.008 | 1.004 | 0.997-1.010 | 1.005 | 0.995-1.015 | 0.983 | 0.958-1.010 |
|  | 3 | 0.996 | 0.986-1.007 | 1.009 | 1.002-1.016 | 1.006 | 0.999-1.013 | 1.000 | 0.989-1.011 | 1.006 | 0.980-1.033 |
|  | 4 | 0.990 | 0.980-1.001 | 1.001 | 0.994-1.008 | 1.003 | 0.996-1.010 | 0.996 | 0.985-1.007 | 1.017 | 0.990-1.044 |
| **I20 – I25** | 0 | 0.998 | 0.980-1.016 | 0.987 | 0.973-1.000 | 0.995 | 0.983-1.007 | 1.005 | 0.986-1.024 | 1.033 | 0.989-1.078 |
|  | 1 | 0.985 | 0.967-1.003 | 0.999 | 0.987-1.012 | 0.998 | 0.986-1.009 | 1.006 | 0.987-1.025 | 1.003 | 0.959-1.049 |
|  | 2 | 0.989 | 0.971-1.008 | 1.003 | 0.991-1.015 | 0.999 | 0.988-1.010 | 1.009 | 0.992-1.026 | 1.000 | 0.957-1.046 |
|  | 3 | 0.991 | 0.973-1.009 | 1.000 | 0.988-1.013 | 0.998 | 0.986-1.010 | 0.995 | 0.976-1.014 | 1.016 | 0.972-1.063 |
|  | 4 | 1.002 | 0.983-1.021 | 1.001 | 0.989-1.014 | 0.997 | 0.985-1.009 | 0.991 | 0.970-1.012 | 0.997 | 0.952-1.045 |
| **I44 – I50** | 0 | 1.020 | 1.008-1.032 | 1.000 | 0.992-1.009 | 0.994 | 0.985-1.002 | 1.007 | 0.996-1.019 | 0.981 | 0.949-1.013 |
|  | 1 | 1.008 | 0.996-1.020 | 1.006 | 0.997-1.014 | 0.999 | 0.991-1.007 | 1.002 | 0.991-1.014 | 0.989 | 0.958-1.022 |
|  | 2 | 0.987 | 0.975-1.000 | 1.000 | 0.991-1.009 | 1.007 | 0.998-1.015 | 1.003 | 0.990-1.015 | 0.975 | 0.943-1.007 |
|  | 3 | 0.999 | 0.987-1.011 | 1.013 | 1.004-1.021 | 1.010 | 1.002-1.018 | 1.002 | 0.989-1.015 | 1.001 | 0.969-1.034 |
|  | 4 | 0.985 | 0.973-0.998 | 1.000 | 0.992-1.009 | 1.006 | 0.998-1.015 | 0.997 | 0.985-1.010 | 1.026 | 0.994-1.059 |
| **I48** | 0 | 1.023 | 1.005-1.040 | 0.991 | 0.978-1.004 | 0.986 | 0.974-0.998 | 1.007 | 0.991-1.024 | 0.988 | 0.943-1.036 |
|  | 1 | 1.016 | 0.999-1.034 | 0.999 | 0.986-1.012 | 0.992 | 0.980-1.004 | 1.006 | 0.991-1.022 | 1.001 | 0.956-1.049 |
|  | 2 | 0.990 | 0.973-1.008 | 1.003 | 0.990-1.016 | 1.003 | 0.991-1.016 | 1.002 | 0.985-1.019 | 1.003 | 0.958-1.052 |
|  | 3 | 0.998 | 0.980-1.016 | 1.009 | 0.996-1.023 | 1.002 | 0.989-1.015 | 1.007 | 0.990-1.025 | 1.022 | 0.976-1.071 |
|  | 4 | 0.995 | 0.977-1.013 | 0.999 | 0.986-1.012 | 1.003 | 0.989-1.016 | 1.001 | 0.984-1.018 | 1.035 | 0.988-1.085 |
